# Supplementary material for: Brain-to-brain coupling forecasts future joint action outcomes
Source: iScience. 2024 Aug 23;27(9):110802. doi: 10.1016/j.isci.2024.110802 (PMC11407023; doi:10.1016/j.isci.2024.110802)
Supplement: Document S1. Figures S1–S5 and Table S1 [file mmc1.pdf]

iScience, Volume *27*

Supplemental information

Brain-to-brain coupling forecasts future joint action outcomes

Roksana Markiewicz, Katrien Segaert, and Ali Mazaheri

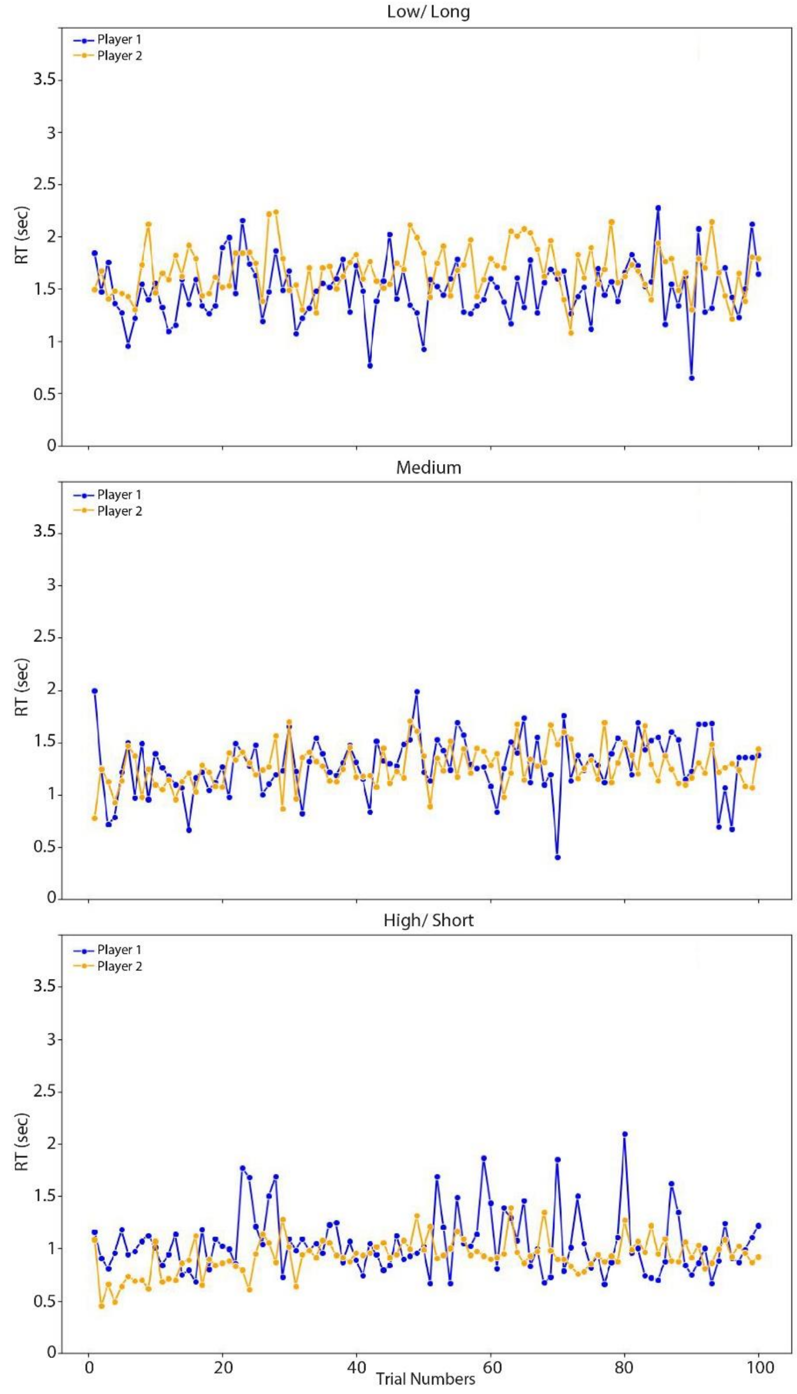

Figure S1. Response times of player 1 (blue) and player 2 (orange) for pair one as an example in function of trial number (note that the trial numbers are dummy coded due to the experimental design, wherein the conditions were interleaved and randomised for each pair, and thus these represent the duration of the experiment rather than the real trial number) for each condition (*top row*: low, *middle row*: medium, and *bottom row*: high). A comprehensive set of similar figures (for other pairs) can be found on: https://osf.io/ct8jb/. We also provide the code used for plotting along with dataset itself, enabling users to adjust the axis limits as needed, particularly in case of outlying responses. *Related to Figure 2 and Table 1 and Table 2.*

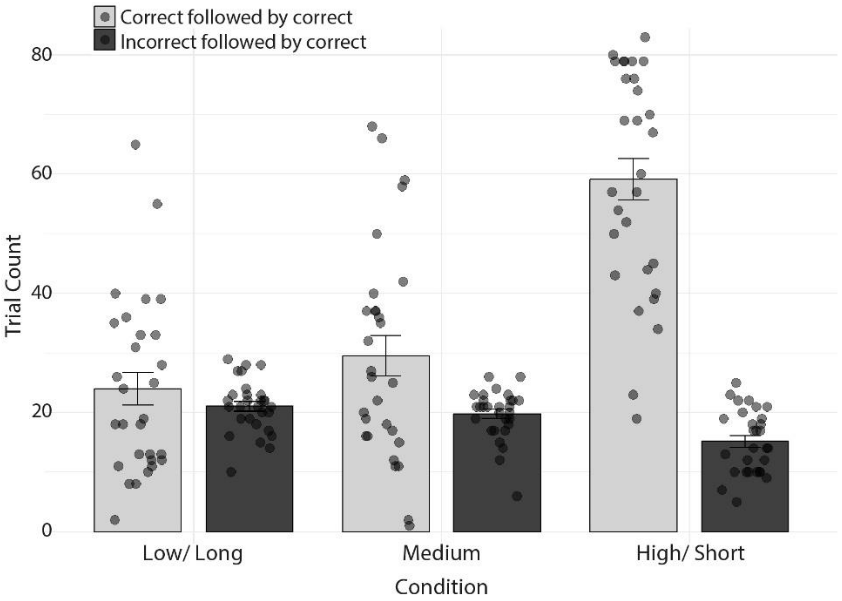

Figure S2. The average frequency of trials where successful cooperation is followed by another successful cooperation (light grey) compared to trials where unsuccessful cooperation is followed by successful cooperation (dark grey) across each condition type (long, medium, and short). Individual data points are represented by black dots, and error bars indicate the standard error. We used a 2 (trial type: Correct followed by correct vs. incorrect followed by correct) x 3 (condition: short, medium, and long) repeated measures ANOVA to compare the frequency of trials where a successful cooperation was followed by another successful trial against those where an unsuccessful cooperation was followed by a successful one for each condition type (short, medium, and long). Overall, we found that participants were more likely to successfully cooperate if the previous trial (of the same type) was also successful. We found that there was a significant main effect of trial type, $F(1, 28) = 40.23$, $p < .001$, revealing that there was a greater frequency of correct followed by correct trials ($M=37.5$, $SE=2.47$) compared to incorrect followed by correct trials ($M=18.6$, $SE=0.565$). There was also a significant main effect of condition $F(1.65, 46.20) = 76.26$, $p < .001$, with a post hoc one-way ANOVA showing that pairs were more likely to successfully cooperate following a previous successful trial compared to when the previous trial was unsuccessful in the short (M difference = 43.93, $F(1, 28) = 99.90$, $p < .001$) and medium (M difference = 9.76, $F(1, 28) = 7.64$, $p = .010$) conditions, but this was not observed in the long condition (M difference = 2.93, $F(1, 28) = 1.05$, $p = .314$). Further, the 2 x 3 repeated measures ANOVA revealed a significant interaction between trial type and condition, $F(1.58, 44.23) = 71.91$, $p < .001$. The results from pairwise comparisons are displayed in Table S1. *Related to Figure 2.*

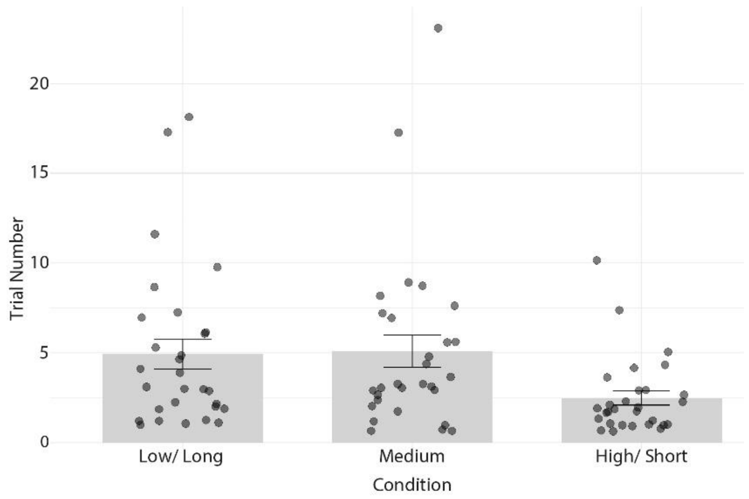

Figure S3. The average trial number at which the first successful cooperation occurred for each condition type (long, medium, and short). Individual data points are represented by black dots, and error bars indicate the standard error. We examined the differences in the trial numbers of the first successful cooperation among the different conditions (long, medium, and short) using a one-way ANOVA. We found a significant effect of condition on the trial number at which the first successful cooperation occurred ($F(2, 84) = 3.85$, $p = 0.025$). Post-hoc Tukey tests indicated a significant difference between the medium and short conditions, showing that the first instance of successful cooperation occurred earlier in the short condition compared to the medium condition ($p = .039$). There was a marginal effect when comparing the long and short conditions, with the first instance of successful cooperation tending to occur earlier in the short condition compared to the long condition ($p = .059$). No significant difference was found between the long and medium conditions ($p = .985$). *Related to Figure 2.*

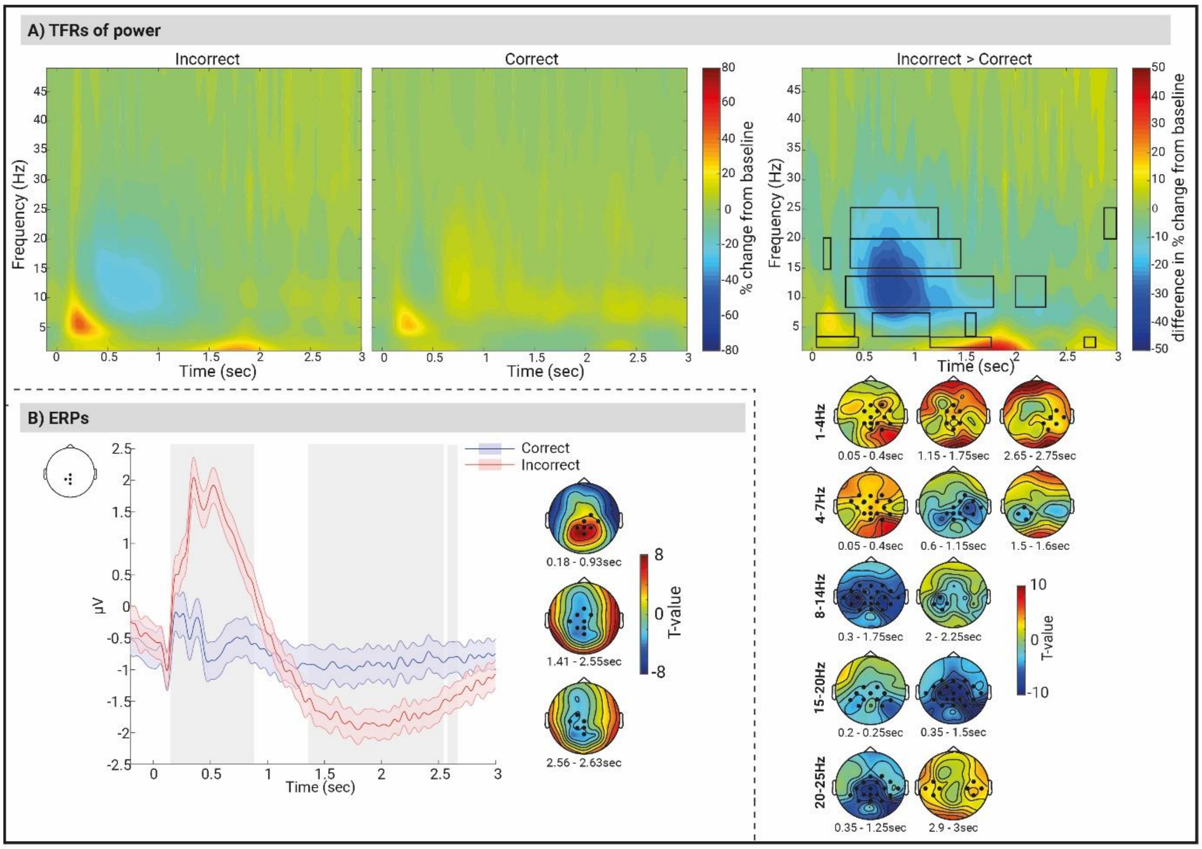

Figure S4. TFR of power and ERPs condition comparisons (incorrect vs. correct) locked to the onset of feedback. Related to Figure 3. (A) TFRs of power (at the Cz electrode) for incorrect trials (left), correct trials (middle), and the condition differences (i.e., incorrect – correct) (right) averaged over all individuals (N = 59), locked to the onset of feedback text. Headplots illustrate the clusters of electrodes that show the most pronounced mean condition difference. Black rectangles indicate significant condition differences ($p < 0.05$, cluster corrected). Firstly, we observed significant condition differences ($p = .003$ and $p < .001$ respectively) in theta and delta activity maximal over the central and centro-parietal channels. Specifically, theta (and delta) power was stronger right after the onset of the feedback text in the incorrect condition until around 0.4sec compared to the correct condition. It has been previously proposed that mid-frontal theta activity serves as a neural indicator of monitoring ongoing actions and signalling unfavourable action outcomes. This in turn results in adaptive response and modulated activity due to committed errors [1,2]. Further, there was a significant condition difference ($p < .001$), with greater delta power in the incorrect vs. correct condition. This effect corresponded to a cluster maximal over the central electrodes that stretched between 1.15 to 1.75sec post feedback text onset. We consider this effect to be a representation of the sustained ERP in the similar time window. Finally for the delta band, we found significantly more pronounced activity in the incorrect compared to the correct condition following the feedback onset ($p = .006$). This effect was most prominent between 2.65sec and 2.75sec post feedback text onset and maximal

over right centro-temporal channels. Further, in the theta band, there was a more sustained theta attenuation in the incorrect compared to the correct condition ($p < .001$). This effect was maximal over centro-parietal and parietal sites and most prominent between 0.6 to 1.15sec post feedback text onset. We take this as spectral leakage from the effect seen in the alpha band, see below for the interpretation of this effect. Lastly for the theta band, we observed a condition effect that was maximal over temporal electrodes and left lateralised ($p = .039$). Specifically, there was a reduction in theta power in the incorrect condition relative to the correct condition corresponding to a cluster that stretched between 1.5 and 1.6sec post feedback onset. We also found significant condition differences in alpha activity. Firstly, there was a greater alpha power decrease in the incorrect condition relative to the correct condition ($p < .001$). This effect corresponded to a cluster that extended between 0.3 to 1.75sec post feedback and was maximal over central electrodes. We interpret this post error alpha decrease as stronger engagement, greater resource allocation and active processing to prevent future errors. This enhanced and more alert cognitive state optimises visual processing and better motor system coordination for future trials [1]. We also found that there was a greater alpha power increase in the correct condition relative to the incorrect condition ($p = .031$). This effect corresponded to a cluster that spread between 2 to 2.25sec and was maximal over left temporal channels.

We found a significant condition effect in the low beta band ($p =.024$). This corresponded to a transient time window of .2 to .25sec post feedback and was maximal over centro-parietal electrodes. Further, there was a prolonged opposite pattern of low beta ($p < .001$) and high beta ($p < .001$) activity between the conditions. The incorrect condition yielded a prolonged low beta decrease, whereas the correct condition showed a prolonged low beta increase. This effect corresponded to a cluster that spanned from .35 to 1.5sec and was prominent all over the scalp. The beta power increase following correct responses and thus feedback is likely reflecting the requirement for strategy maintenance by facilitating the reinforcement of the current motor and cognitive strategy [3–7]. Lastly, we found a transient condition effect ($p = .013$) in the high beta band that extended between 2.9 and 3sec post feedback. Specifically, there was a greater high beta power increase in the incorrect compared to the correct condition, maximal over bilateral temporal sites.

(B) Feedback locked averaged ERPs produced by correct (blue) and incorrect (red) trials in the timing task averaged over all individuals ($N = 59$). The ERP waveforms show averaged ERPs across the overlapping (across the three time windows of the significant between condition effects) electrode clusters (Cz, CP1, Pz, and CPz) that indicate the maximal condition difference (a schematic view of these electrodes is shown in the top right corner). The shaded areas around the ERP waves represent standard error. The grey rectangles represent time windows of the significant between condition differences. The black dots in the headplots illustrate the clusters of electrodes showing most pronounced mean condition differences for each time window. The cluster-based permutation tests revealed that there was a significant effect of condition, specifically the incorrect condition was

associated with a greater positivity compared to the correct condition ($p < .001$). This effect corresponded to a cluster that spanned from .178 to .926sec and was maximal over centro-parietal electrodes. Secondly, we found another condition effect, where the incorrect trials yielded a sustained negativity ERP relative to the correct trials ($p < .001$, $p = .034$). This corresponded to clusters that extended from 1.406 to 2.554sec as well as 2.562 to 2.634sec respectively, most prominent over centro-parietal channels. Previous research has proposed that the localization of error-related negativity (ERN) and theta activity overlap, suggesting a shared involvement in error monitoring processes [1]. Additionally, it has been suggested that the ERN serves not only as an error detection mechanism but also predicts the extent of learning from errors [8]. Consequently, we interpret this ERP effect playing a role in error monitoring and adjustment for future actions.

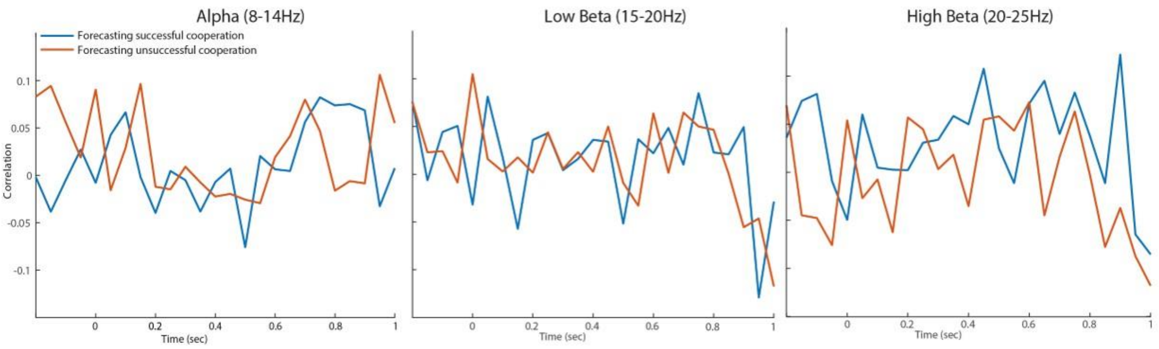

Figure S5. Forecasting successful (blue) and unsuccessful (orange) joint action outcomes on next consecutive trials based on the correlation values (Fisher Z) of oscillatory power coupling between player 1 and player 2 after the onset of feedback text in the failed cooperation condition. Line plots are representing the correlation (Fisher Z) values averaged over the electrodes from all over the scalp (the averaged electrodes are those that show maximal condition differences between correct and incorrect trials in each frequency band; however, note that the electrodes are different for each frequency band; see Fig. S4A) in the alpha (8-14Hz; left panel), low beta (15-20Hz; middle panel), and high beta (20-25Hz; right panel) frequency bands. Note that there are no significant condition differences in either of the frequency bands ($p < .0.05$, cluster corrected). *Related to Figure 4.*

Table S1. *Post-hoc multiple comparisons (with Bonferroni correction) for the frequency of trials where a successful cooperation was followed by another successful trial (Correct followed by Correct) against those where an unsuccessful cooperation was followed by a successful trial (Incorrect followed by Correct) between conditions (short, medium, and long). Adjusted p indicates the p-value after Bonferroni correction. Related to Figure 2.*

Trial type	Condition(1)	Condition(2)	N(1)	N(2)	t	df	P	Adj. p
Correct followed by Correct	Long	Medium	29	29	-2.60	28	.015	.045
Correct followed by Correct	Long	Short	29	29	-10.30	28	<.001	<.001
Correct followed by Correct	Medium	Short	29	29	-9.20	28	<.001	<.001
Incorrect followed by Correct	Long	Medium	29	29	1.68	28	.105	.315
Incorrect followed by Correct	Long	Short	29	29	4.65	28	<.001	<.001
Incorrect followed by Correct	Medium	Short	29	29	4.13	28	<.001	<.001

1. Mazaheri, A., Nieuwenhuis, I.L.C., Van Dijk, H., and Jensen, O. (2009). Prestimulus alpha and mu activity predicts failure to inhibit motor responses. Hum Brain Mapp *30*, 1791–1800. https://doi.org/10.1002/hbm.20763.

2. Beldzik, E., Ullsperger, M., Domagalik, A., and Marek, T. (2022). Conflict- and error-related theta activities are coupled to BOLD signals in different brain regions. Neuroimage *256*, 119264. https://doi.org/10.1016/j.neuroimage.2022.119264.

3. Engel, A.K., and Fries, P. (2010). Beta-band oscillations-signalling the status quo? Curr Opin Neurobiol *20*, 156–165. https://doi.org/10.1016/j.conb.2010.02.015.

4. Cohen, M.X., Elger, C.E., and Ranganath, C. (2007). Reward expectation modulates feedback-related negativity and EEG spectra. Neuroimage *35*, 968–978. https://doi.org/10.1016/j.neuroimage.2006.11.056.

5. Yeung, N., Botvinick, M.M., and Cohen, J.D. (2004). The neural basis of error detection: Conflict monitoring and the error-related negativity. Psychol Rev *111*, 931–959. https://doi.org/10.1037/0033-295X.111.4.931.

6. Marco-Pallares, J., Cucurell, D., Cunillera, T., Garcia, R., Andres-Pueyo, A., Munte, T.F., and Rodriguez-Fornells, A. (2008). Human oscillatory activity associated to reward processing in a gambling task. Neuropsychologia *46*, 241–248. https://doi.org/10.1016/j.neuropsychologia.2007.07.016.

7. Marco-Pallares, J., Cucurell, D., Cunillera, T., Krämer, U.M., Càmara, E., Nager, W., Bauer, P., Schüle, R., Schöls, L., Münte, T.F., et al. (2009). Genetic Variability in the Dopamine System (Dopamine Receptor D4, Catechol-O-Methyltransferase) Modulates Neurophysiological Responses to Gains and Losses. Biol Psychiatry *66*, 154–161. https://doi.org/10.1016/j.biopsych.2009.01.006.

8. Frank, M.J., Woroch, B.S., and Curran, T. (2005). Error-related negativity predicts reinforcement learning and conflict biases. Neuron *47*, 495–501. https://doi.org/10.1016/j.neuron.2005.06.020.
